# The non‐specific lethal (NSL) complex at the crossroads of transcriptional control and cellular homeostasis

**DOI:** 10.15252/embr.201847630

**Published:** 2019-06-03

**Authors:** Bilal N Sheikh, Sukanya Guhathakurta, Asifa Akhtar

**Affiliations:** ^1^ Max Planck Institute for Immunobiology and Epigenetics Freiburg im Breisgau Germany; ^2^ Faculty of Biology Albert Ludwig University of Freiburg Freiburg Germany

**Keywords:** epigenetics, HAT, KANSL1, Koolen de‐Vries, transcription, Chromatin, Epigenetics, Genomics & Functional Genomics

## Abstract

The functionality of chromatin is tightly regulated by post‐translational modifications that modulate transcriptional output from target loci. Among the post‐translational modifications of chromatin, reversible ε‐lysine acetylation of histone proteins is prominent at transcriptionally active genes. Lysine acetylation is catalyzed by lysine acetyltransferases (KATs), which utilize the central cellular metabolite acetyl‐CoA as their substrate. Among the KATs that mediate lysine acetylation, males absent on the first (MOF/KAT8) is particularly notable for its ability to acetylate histone 4 lysine 16 (H4K16ac), a modification that decompacts chromatin structure. MOF and its non‐specific lethal (NSL) complex members have been shown to localize to gene promoters and enhancers in the nucleus, as well as to microtubules and mitochondria to regulate key cellular processes. Highlighting their importance, mutations or deregulation of NSL complex members has been reported in both human neurodevelopmental disorders and cancer. Based on insight gained from studies in human, mouse, and *Drosophila* model systems, this review discusses the role of NSL‐mediated lysine acetylation in a myriad of cellular functions in both health and disease. Through these studies, the importance of the NSL complex in regulating core transcriptional and signaling networks required for normal development and cellular homeostasis is beginning to emerge.

**Glossary**
ANKRD2: ankyrin repeat domain 2
ARL17A: ADP ribosylation factor like GTPase 17A
BAH: bromo adjacent homology
bp: base pair
*C. elegans*: *Caenorhabditis elegans* (nematode)
ChIP: chromatin immunoprecipitation
*D. melanogaster*: *Drosophila melanogaster* (fruit fly)
*D. rerio*: *Danio rerio* (zebrafish)
EHMT2: euchromatic histone lysine N‐methyltransferase 2
ESC: embryonic stem cells
GFAP: glial fibrillary acidic protein
HAT: histone acetyltransferase
HCF1: host cell factor 1
HDAC: histone deacetylase
Hox: homeobox
KANSL: KAT8‐associated non‐specific lethal
KAT: lysine acetyltransferase
KAT8: lysine acetyltransferase 8; also known as MOF
KDAC: lysine deacetylase; also known as HDAC
KdV: Koolen de‐Vries
MCRS: microspherule protein 1
MBT: malignant brain tumor protein
MBD‐R2: methyl‐CpG‐binding domain protein R2
MEFs: mouse embryonic fibroblasts
MLL: mixed lineage leukemia
MOF: males absent on the first
MOZ: monocytic leukemia zinc finger protein; also known as KAT6A
MSL: male‐specific lethal
MYST: MOZ, YBF2, SAS2, and TIP60
NPC: neural progenitor cell
NSCLC: non‐small‐cell lung carcinoma
NSL: non‐specific lethal
NUP98: nucleoporin 98
NuRD: nucleosome remodeling and deacetylase
NURF: nucleosome remodeling factor
OGT: UDP‐N‐acetylglucosamine—peptide N‐acetylglucosaminyltransferase
OXPHOS: oxidative phosphorylation
PHD: plant homeodomain
PHF20: plant homeodomain finger protein 20
POU5FI: POU class 5 homeobox 1
RBBP5: retinoblastoma‐binding protein 5
Rheb: Ras homolog enriched in brain
RUNX2: Runt‐related transcription factor 2
SET: Su(var)3‐9, Enhancer‐of‐zeste and Trithorax
TET: Ten‐eleven translocation
TOP1: DNA topoisomerase 1
TPX2: targeting protein for Xklp2
TUBB3: tubulin beta‐3 chain
WDR5: WD Repeat containing domain 5
YEATS: Yaf9 ENL AF9 Taf14 Sas5

## Introduction

Each human cell contains around 2 m of DNA, which must be packaged into a single nucleus that on average is 6 μm in diameter. The cell achieves this feat by organizing and packaging DNA with the help of histone proteins, which together make up chromatin. The fundamental unit of chromatin is the nucleosome, which contains approximately 146 bp of double‐stranded DNA wrapped around a histone octamer, composed of two of each histones H2A, H2B, H3, and H4 1. Together, interconnected nucleosomes give chromatin a “beads‐on‐a‐string” appearance. Rather than being homogenously distributed in the nucleus, chromatin is segregated into functional domains that closely regulate the transcriptional activity of the associated DNA 2. Chromatin structure is dynamic, and changes in chromatin are associated with cell fate specification during development 3, 4, 5 and maintenance of cellular homeostasis in the face of cellular stresses 6, 7.

Chromatin structure is regulated and modified by a combination of post‐translational modifications that are widespread on DNA and histones. Over 130 post‐translational modifications of histone proteins have been described to date. These span acetylation, methylation, ubiquitination, and phosphorylation among others 8. Unique combinations of histone post‐translational modifications have been proposed to constitute the “histone code” 9, 10, which signals the functionality of the associated region of chromatin. Post‐translational modifications of chromatin can affect the function of chromatin in at least three ways:

1. Modifying the interaction between DNA and histones 11.
2. Modulating interactions between neighboring histones 12, 13, and thereby local chromatin structure.
3. Forming docking sites for the recruitment of chromatin‐modifying complexes 14.

The post‐translational modifications of DNA and histones are laid down by multi‐subunit chromatin‐modifying complexes, which are recruited and subsequently act at specific regions of chromatin. Highlighting their importance, mutations in members of chromatin‐modifying complexes typically result in human developmental disorders or cancer 15, 16.

Among the range of post‐translational modifications, reversible ε–lysine acetylation is particularly prominent within cells 17. Histones associated with transcriptionally active chromatin are widely decorated with acetylated lysines 18. Furthermore, over 2,000 cellular proteins have been identified by mass spectrometry analysis to possess at least one acetylated ε–lysine residue 17. Besides histones, mitochondrial proteins and metabolic enzymes show high and dynamic levels of lysine acetylation 19. The acetylation reaction utilizes acetyl‐CoA, an intermediate metabolite generated from metabolic pathways such as aerobic glycolysis and fatty acid oxidation, to catalyze the acetylation reaction 20. Given that cellular acetyl‐CoA levels correlate with the energy state of the cell 21, protein acetylation levels link the metabolic status and epigenetic landscape of cells 22. While the acetylation reaction can occur non‐enzymatically under certain conditions such as alkaline pH 23, which is commonly found in the mitochondria, much of the acetylation in the cytoplasm and nucleus is thought to be enzymatically catalyzed. Enzymatic acetylation of lysine residues is mediated by KATs, which possess an acetyl‐CoA binding site and can transfer the acetyl group from acetyl‐CoA to the Nε–residue of lysines 20. The opposing deacetylation reaction is undertaken by KDACs (also known as HDACs) 24.

Acetylation of chromatin and its importance for transcription have been particularly well studied. Among the acetylation marks found on chromatin, histone 4 lysine 16 acetylation (H4K16ac) is particularly notable for its ability to decompact chromatin structure 12, 13. H4K16ac is catalyzed by the MYST‐family KAT MOF (also known as KAT8) 25, which is conserved from *Drosophila* to human 25, 26, 27, 28, 29, 30. The function of MOF has been best characterized in the fruit fly *D. melanogaster*, where it upregulates transcription twofold from the male X‐chromosome to mediate sex‐based dosage compensation (for review see 31, 32, 33). MOF undertakes sex‐based dosage compensation in *D. melanogaster* in the context of the *male‐specific lethal* (MSL) complex. In addition to the MSL complex, MOF has been found as the catalytic member of the highly conserved *non‐specific lethal* (NSL) complex 34. Compared with the MSL complex, functions of the NSL complex remain enigmatic and have only recently started to be determined. The NSL complex appears to be widespread within cells, acts broadly, and controls pathways that are critical for organismal development and cellular homeostasis. This review will focus on the functions of the NSL complex in transcription, its role in the mitochondria and in cell division, as well as the importance of the NSL proteins in human disease.

## Two independent MOF complexes—MSL and NSL

**Figure 1:**
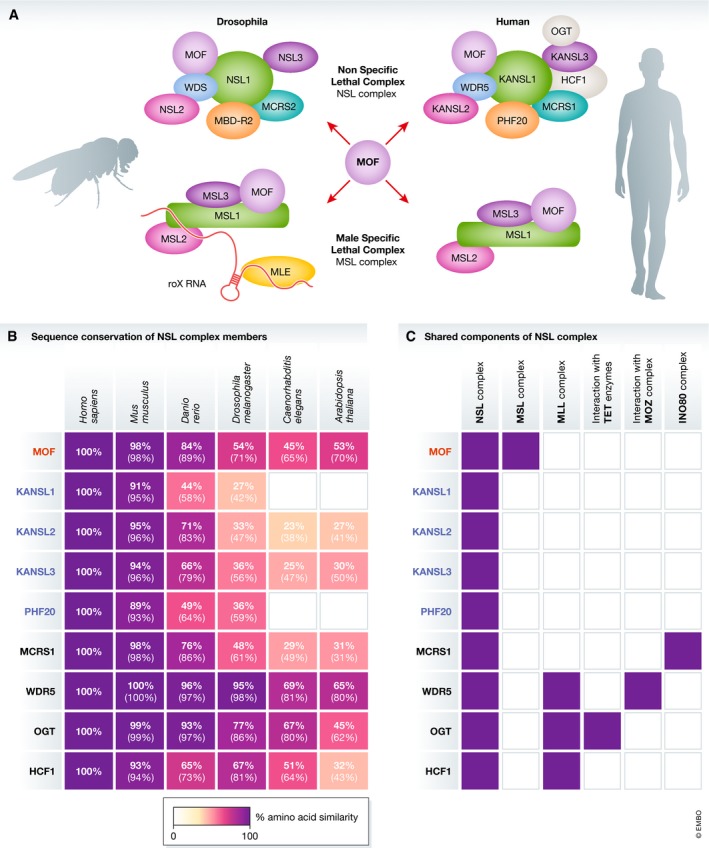
Characteristics of NSL complex proteins (A) The two MOF‐containing complexes—the NSL and MSL complexes. The NSL and MSL complex components in the two best studied models, *D. melanogaster* and human, are depicted. (B) Conservation of the NSL complex through evolution. The heatmap indicates the level of amino acid similarity in the *conserved domains* of NSL complex members. The percentages written over the heatmap indicate the precise amino acid identity in the conserved domains, with the percentage similarity is provided in the parenthesis. The human NSL protein information was extracted from the NCBI protein database followed by *blast* of the whole protein sequence to determine the identity of NSL complex orthologs and amino acid conservation. (C) Core versus shared members of the NSL complex. MOF, the catalytic subunit of the NSL complex, is also found in the MSL complex 40. OGT also interacts with the TET enzymes TET1‐3 that modify the DNA 5‐methyl‐cytosine base to 5‐hydroxymethyl‐cytosine 104, 105, 106, 107, as well as the MLL/SET complexes 108. In addition to the NSL complex, WDR5 also associates with the MLL trithorax 109 and the MOZ acetyltransferase 110 complexes. HCF1 is shared with the MLL trithorax complex 108, 109, 111, while MCRS1 co‐immunoprecipitates both NSL and INO80 complex members 40. In contrast, KANSL1, KANSL2, KANSL3 and PHF20 have only been identified as part of the NSL complex to date.

MOF forms the catalytic core of two mutually exclusive complexes that are conserved from *Drosophila* to mammals (Fig 1A). MOF was first established as part of the MSL complex over two decades ago. Seminal studies in *D. melanogaster* showed that the MSL complex is required for the twofold upregulation of H4K16ac and gene transcription from the single male X‐chromosome 25, 26, 28. This function of the MSL complex is necessary to balance the gene expression from a single male X‐chromosome to expression from two X‐chromosomes in females. While the sex‐based dosage compensation system in mammals is distinct from *Drosophila*, the MSL complex has recently been implicated in regulating X‐chromosome inactivation during differentiation of female mouse ESCs 35. In contrast to *Drosophila*, one of the two X‐chromosomes in females is inactivated to balance gene dosage between males and females in mammals. In mouse ESCs, the MOF‐MSL complex binds to the *Tsix*/*Xist* locus 35, which encodes for the *Tsix* and *Xist* long non‐coding RNAs that are essential for X‐chromosome inactivation. In the absence of *Msl1* or *Msl2*,* Tsix* levels are reduced, *Xist* shows a concomitant increase in expression, and chaotic X‐chromosome inactivation is observed in differentiating ESCs 35. The importance of the MSL complex in mediating transcriptional regulation seems to extend beyond sex‐based dosage compensation. Recent work has established that the MSL complex drives H4K16ac and transcription of highly conserved developmental genes in *D. melanogaster* and mouse 36. Consistently, loss of just one allele of *MSL3* leads to human developmental disorders typified by intellectual disability and developmental delay 37. While the recruitment mechanisms for the MSL complex to the *D. melanogaster* male X‐chromosome are well studied, future studies are needed to determine how the MOF‐MSL complex is recruited to developmentally important genes on autosomes. The recruitment mechanism of the mammalian MSL complex is likely to be unique, as the non‐coding roX RNAs that recruit the *Drosophila* MSL complex to the male X‐chromosome are not found in the mammalian MSL complex 38.

The first indications that MOF could interact with additional proteins beyond the MSL complex date back to 2005 28, 39. In 2006, through systematic purifications and mass spectrometry analyses in *D. melanogaster* larvae, SL2 cells, and human HeLa cells, Akhtar and co‐workers identified the full complement of NSL complex members (Table 1) 34, which was also later independently confirmed by Conaway and colleagues 40. The non‐specific lethal (NSL) complex got its name due to the phenotypes of NSL‐knockout *D. melanogaster* flies. In contrast to depletion of the MSL complex members, which induces stronger phenotypes in males due to dosage compensation defects, depletion of the NSL complex members adversely affected the survival of both male and female flies 34.

**Table 1:** NSL complex members across species

Human	Mouse	Zebrafish	Fly	Worm	*A. thaliana*
MOF (KAT8)	MOF (KAT8)	kat8	MOF	MYS‐2	HAM1^a^
KANSL1	KANSL1	kansl1	NSL1	–	–
KANSL2	KANSL2	kansl2	NSL2 (DGT1)	SUMV‐1	INO80 complex subunit D‐like^a^
KANSL3	KANSL3	kansl3	NSL3 (RCD1)	SUMV‐2	α/β hydrolase superfamily protein^a^
PHF20	PHF20	phf20b	MBD‐R2	–	–
MCRS1	MCRS1	mcrs1	MCRS2 (RCD5)	MCRS‐1	FHA domain containing protein^a^
WDR5	WDR5	wdr5	WDS	WDR‐5.1	Transducin^a^
OGT	OGT	ogt	SXC^a^	OGT‐1	TPR‐like superfamily protein^a^
HCF1	HCF1	hcf1	HCF^a^	HCF‐1	Galactose oxidase/kelch repeat superfamily protein^a^

Human—*Homo sapiens*; Mouse—*Mus musculus*; Zebrafish—*Danio rerio*; Fly—*Drosophila melanogaster*; worm—*Caenorhabditis elegans*;* A. thaliana*—*Arabidopsis thaliana*.

^a^These orthologs have been identified through sequence conservation. Whether they are part of the NSL complex remains to be determined.

## Constituents of the NSL complex

The NSL complex is highly conserved, and NSL orthologs have been identified in a range of organisms including *C. elegans* 41, 42 and *D. rerio* (Fig 1B). There are four core members of the NSL complex, KANSL1 (also referred to as MSL1v1 28, 39, 43), KANSL2, KANSL3, and PHF20, which have only been identified as part of the NSL complex to date 40 (Fig 1C). In contrast, other members of the NSL complex are shared with other chromatin‐modifying complexes (Fig 1C).

**Figure 2:**
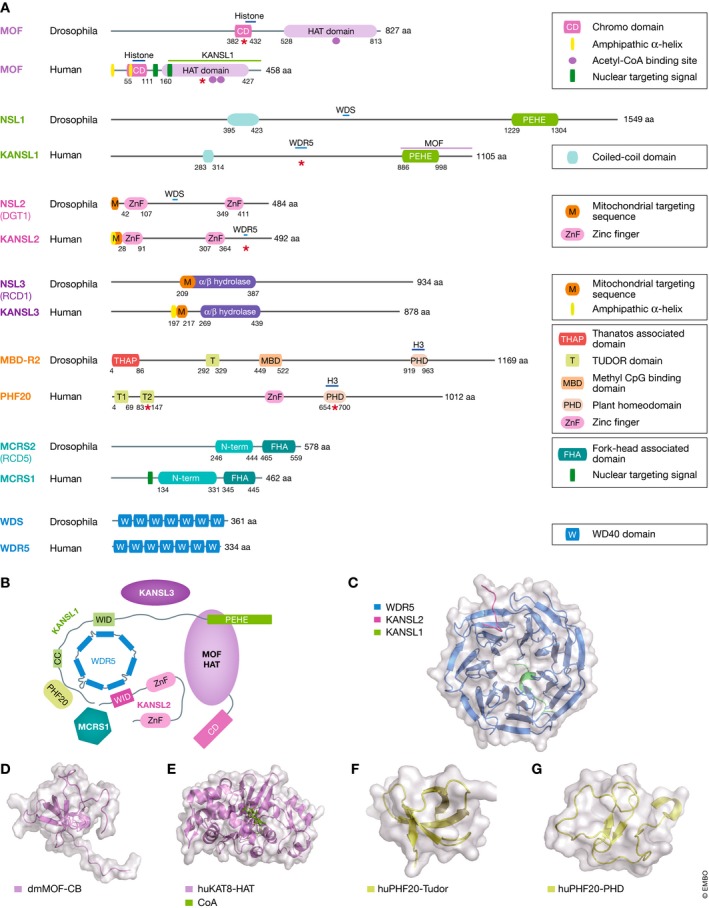
Domain organization and structure of NSL complex members (A) Schematic representation of protein domain organization of human (hu) and *D*. *melanogaster* (dm) NSL complex members based on NCBI annotations. Numbers on either side of the respective domain represent its start and end amino acid position. The horizontal bars denote the binding region of the protein indicated above it. The red stars indicate the protein domains of the structures demonstrated in panels (C‐G). (B) Summary model of human NSL complex based on structural and protein interaction studies 44. (C) Ribbon representation of the crystal structure of a KANSL1, WDR5, and KANSL2 sub‐complex (PDBID 4CY2) 44; (D) NMR structure of the chromobarrel domain of *D. melanogaster* MOF (PDBID 2BUD) 112; (E) X‐ray diffraction structure of the HAT domain of human MOF (PDBID 2PQ8); (F) X‐ray structure of the Tudor domain of PHF20 (PDBID 3QII) 113; (G) PHD domain of PHF20 (PDBID 5TAB) 57.

Recent biochemical and structural studies have started to shed light on how the NSL complex is organized. Both in *D. melanogaster* and humans, KANSL1 is unstructured and thought to act as the major scaffolding protein within the NSL complex 44 (Fig 2A and B). KANSL1 binds to MOF via its PEHE domain, while interacting with PHF20 and MCRS1 via its N‐terminus (Fig 2A–C). Reminiscent of MSL1 in the MSL complex, the interaction of MOF with KANSL1 is required to potentiate the catalytic activity of the NSL complex toward H4K16 and p53‐K120, at least in extracellular acetylation assays 40, 43. Structural work has demonstrated that arginine 592 in human KANSL1 and arginine 721 in *D. melanogaster* NSL1 mediate their interaction with WDR5/WDR 44. Substitution of just this one arginine to an alanine is sufficient to hinder the KANSL1‐WDR5 interaction. While KANSL1 interacts with one side of WDR5, KANSL2 binds to the opposing side via the L411 and V413 residues. Interestingly, the interaction of KANSL1 and KANSL2 with WDR5 is reminiscent of the interaction of WDR5 with MLL and RbBP5, suggesting that the WDR5 subunits in the NSL and MLL complexes are distinct. The structural analysis thus provides the molecular basis of how WDR5 engages in mutually exclusive interactions with MLL and KANSL1, thus leading to two independent and distinct MLL and NSL complexes that share WDR5. Since both complexes are promoter‐bound, an interplay between MLL and NSL complexes will be an interesting avenue to explore.

Collaboration between NSL complex members is important for mediating transcriptional activation. The NSL complex member OGT O‐GlcNAcylates both KANSL3 45 and HCF1 46 in immortalized human cells. It has been proposed that OGT‐mediated O‐GlcNAcylation of KANSL3 is required for the stability of KANSL3 within the NSL complex and the subsequent catalytic activity of the complex 45. Consistently, in NSL3‐DNA‐GAL4 tethered luciferase assays, knockdown of *Drosophila mcrs2*,* nsl1,* or *mof* leads to a reduction in NSL3‐mediated luciferase activity 47. In the context of the human SET1/COMPASS complex, OGT‐mediated O‐GlcNAcylation of HCF1 is required for complex stability and methyltransferase activity of SET1/COMPASS at H3K4 46. Whether OGT also O‐GlcNAcylates HCF1 in the context of the NSL complex and how this may affect NSL complex activity remains to be determined.

In addition to the collaboration within the NSL complex, there also appears to be a recruitment hierarchy. Depletion of *mof* in *Drosophila* S2 cells does not adversely affect the stability of other complex members 47. In contrast, *mcrs2*,* nsl1,* or *nsl3* RNAi leads to reduced protein levels of NSL complex partners, suggesting that recruitment into the NSL complex is important for the stability of NSL proteins. Similarly, depletion of *mcrs2* in *Drosophila* leads to reduced binding of MOF, NSL1, and MBD‐R2 at chromatin 47. Furthermore, disruption of the NSL1‐WDS interaction results in lower levels of NSL1 and MBD‐R2 at NSL target genes 44. While more work is required to determine the precise order of NSL protein recruitment to chromatin, these data suggest that incorporation of NSL proteins into the NSL complex is important for their stability. In addition, it is likely that MOF is one of the last components to be recruited into the NSL complex, as MOF depletion does not adversely affect other complex members. It is thus tempting to speculate that both NSL and MSL complexes can form on chromatin and MOF is preferably recruited to one of the two complexes depending on currently unidentified factors. In support of this hypothesis, “free” MOF that is independent of both the MSL and NSL complexes has been reported in mouse ESCs 48, as well as in biochemical assays undertaken in 293T cells 43. Whether MOF has any activity *in vivo* outside of the NSL and MSL complexes is unknown. However, given that MSL1 and KANSL1 stimulate the catalytic activity of MOF 43, it is unlikely that “free” MOF possesses significant catalytic activity in cells. Despite the insights provided by the studies discussed here, precisely how the NSL complex members come together to form a stable complex and are targeted to chromatin remains to be established. Furthermore, factors that promote MOF recruitment into the NSL versus MSL complex remain to be identified.

## NSL complex at chromatin

**Figure 3:**
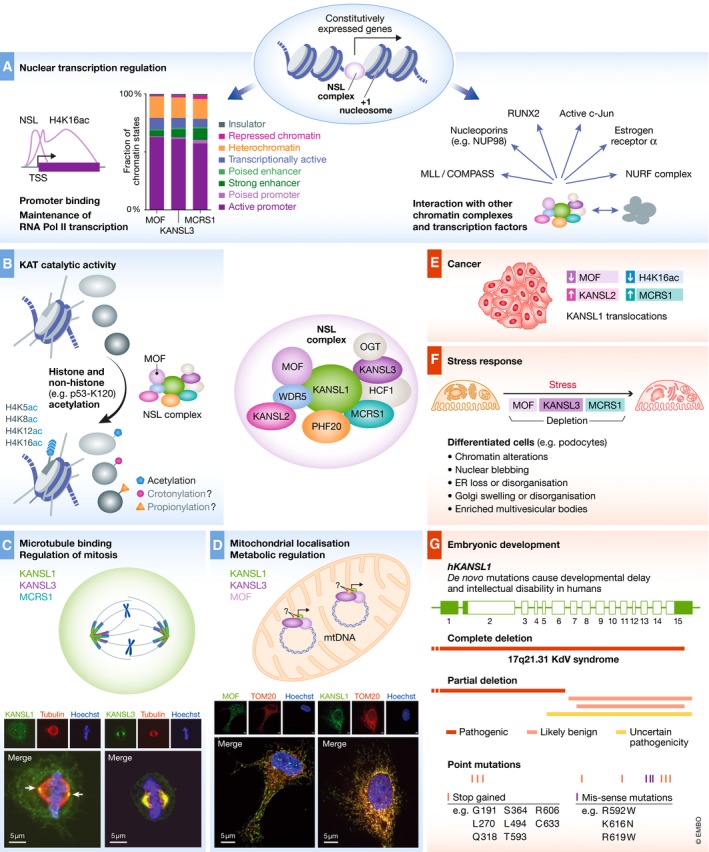
Multiple facets of the NSL complex (A) The NSL complex regulates nuclear gene transcription. In *D. melanogaster,* the NSL complex localizes to gene promoters and maintains the positioning of the +1 nucleosome (middle panel). In addition, the NSL complex acetylates H4K16, which is enriched at promoters as well as gene bodies of transcriptionally active genes (left panel). A similar chromatin‐binding pattern is observed in mammalian cells, whereby MOF, KANSL3, and MCRS1 localize to active gene promoters and to a lesser extent, to enhancers in mouse ESCs. The plot on the left side of panel (A) was generated by apportioning all NSL‐bound regions in ChIP‐seq data from male mouse ES cells 35 into different chromatin states 114. To mediate transcriptional control, the MOF‐NSL complex interacts with other chromatin complexes and transcription factors, allowing for crosstalk and collaboration between different signaling pathways and chromatin regulators (right panel). (B) Besides histones, MOF acetylates non‐histone proteins such as p53 115. This encourages investigation of MOF partner proteins and MOF targets outside of chromatin. Moreover, MOF has been implicated in catalyzing other –acyl chains such as crotonylation and propionylation 116, 117. However, the precise contribution of MOF and its NSL complex members to non‐acetyl acylations needs further investigation. (C) KANSL1, KANSL3, and MCRS1 localize to microtubules during mitosis and are required for the progression of cells through cell division. (D) The NSL complex members MOF, KANSL1, and KANSL3 localize to the mitochondria. This finding has opened up a new horizon for the study of the predominantly chromatin‐associated NSL complex in the regulation of mitochondrial biology. (E) MOF, KANSL1, KANSL2, and MCRS1 are dysregulated in various types of cancer, and the misregulation of NSL complex members drives tumor pathology and aggressiveness. (F) The MOF‐NSL complex is implicated in multi‐organellar stress responses by maintaining key transcriptional networks such as autophagy and DNA repair. (G) Heterozygous deletion or point mutations in human *KANSL1* (adapted from patient entries on DECIPHER GRCh37) cause the KdV syndrome, which is typified by developmental abnormalities and intellectual disability. How the NSL complex regulates development and brain function, however, is an outstanding question.

Consistent with the role of MOF in mediating H4K16 acetylation at chromatin, the NSL complex is enriched in the nucleus. ChIP of NSL complex members in *D. melanogaster* followed by next‐generation sequencing found NSL complex members including NSL1, NSL3, MCRS2, and MBD‐R2 to localize to promoters of over 4,000 genes 47, 49. Analysis of NSL‐bound genes in *D. melanogaster* revealed that NSL proteins are enriched at gene promoters of constitutively active housekeeping genes (Fig 3A, left panel) 47, 49, 50. These NSL target promoters are highly enriched in histone modifications classically associated with transcriptionally active promoters including H4K16ac, H3K9ac, H3K4me2, and H3K4me3 49.

More recently, genome‐wide ChIP profiles have also been generated for the mouse NSL complex members KANSL1 48, KANSL3 35, MCRS1 35, and MOF 35, 48, 51 in ESCs and NPCs. Like *Drosophila*, mammalian MOF‐NSL complex members tend to localize to promoters of transcriptionally active genes 35, 48, 51. While a significant proportion of NSL‐bound genes in ESCs are also housekeeping, the mammalian NSLs are also present at some developmentally important genes and enhancers. Indeed, KANSL3 35 and MOF 52 bind to a subset of active enhancer elements in mouse ESCs and their binding is lost in more differentiated cell types. While the significance of MOF and KANSL3 binding to enhancers in ESCs remains to be determined, MOF appears to be important for maintenance of the Nanog‐mediated pluripotency network in mouse ESCs 51. This function of MOF is likely to be mediated in the context of the NSL complex, as depletion of *Kansl3* but not *Msl1* or *Msl2* leads to the loss of alkaline phosphatase staining and reduced expression of the pluripotency factors Nanog, REX1, and ESRRB in mouse ESCs 35. Consistently, ESC lines could not be established from blastocysts lacking *Mof* 27 or *Phf20* 53, while fibroblasts lacking *Phf20* could not activate pluripotency genes after induction of pluripotency via the Yamanaka factors 53. These cell culture experiments suggest an important role for the NSL complex during the developmental period. Consistently, *Mof*‐knockout mice display early embryonic lethality at around the blastocyst stage 27, 30, while *Phf20*‐knockout mice are highly runted and the majority die around birth 54. However, the *in vivo* role of other NSL complex members during development remains to be established via mouse models, especially in a tissue‐ or cell‐specific manner.

Precisely what mediates the requirement for the NSL complex for transcription remains an open question. It is plausible that the NSL complex binds and primes a subset of genes for activation upon a certain stimulus. Underpinning this hypothesis, MOF has recently been suggested to be important for stress response in kidney podocytes in the context of the NSL complex 55. Conditional deletion of *Mof* in terminally differentiated podocytes, key components of the kidney filtration barrier, had no adverse effect at steady state. However, when conditional *Mof‐*knockout mice were exposed to a mild stressor, they were unable to respond leading to the breakdown of glomerulus structure and kidney failure 55. Comparison of gene expression changes and ChIP profiles suggested that MOF induced the response to cellular stress in podocytes in the context of the NSL complex. In the future, it would be of interest to independently verify the importance of NSL complex members in stress responses.

Given that the main function of MOF is acetylation of H4K16, studies have looked at the ability of MOF to acetylate H4K16 as part of the NSL complex. While extracellular biochemical studies with selected complex members added to MOF have shown NSL complex activity toward H4K16ac 40, studies undertaken in cells and organisms have suggested that the NSL complex maintains only a small proportion of global H4K16ac. While knockdown of *Mof* or *Msl1* in mouse ESCs leads to a global reduction in H4K16ac, no global change in H4K16ac is observed upon shRNA‐mediated knockdown of *Kansl1* or *Kansl3* 35, 48. Similarly, while chromatin binding of NSL1 and MBD‐R2 in *D. melanogaster* shows an overlap with H4K16ac, depletion of *mcrs2* in SL2 cells does not lead to a bulk reduction in H4K16ac levels 47. Quantitative mass spectrometric analysis of histones has also confirmed that *msl1* and *mof* but not *nsl1* depletion lead to reduced bulk H4K16ac in *Drosophila* S2 cells 56. Together, these findings suggest that MOF is more likely to mediate global H4K16ac in the context of the MSL complex. Nevertheless, it is possible that NSL complex depletion leads to a more localized reduction in H4K16ac levels around NSL‐bound promoters. Indeed, Klein and co‐workers found reduced H4K16ac at PHF20 target genes upon *PHF20* knockdown in H1792 lung adenocarcinoma cells 57. In this context, it is also interesting to note that depletion of NSL complex members is associated with reduced levels of RNA polymerase II, TBP, and TFIIB at NSL target promoters in flies 49, suggesting that the NSL complex may also be involved in the recruitment of the transcriptional machinery. In *D. melanogaster*, NSL1 binding shows a strong overlap with members of other core chromatin complexes including the trithorax, chromator, and NURF complexes 50, 58. Thus, it is tempting to speculate that like other KATs such as p300 and CBP 59, the MOF‐NSL complex may also directly acetylate and modulate the activity of chromatin regulators or the transcriptional machinery. Consistently, *Drosophila* MCRS2 is perpetrated to directly interact with and recruit the RNA polymerase II complex to target genes 60. In the future, it will be important to determine the precise extent of NSL activity toward H4K16 *in vivo* as well as the H4K16‐independent targets of the NSL complex in transcription.

## Cooperation between the NSL complex and other chromatin complexes

Individual chromatin complexes do not function on their own, but rather cooperate with other nuclear proteins to fine‐tune transcriptional programs (Fig 3A, right panel). There are two main mechanisms through which chromatin complexes achieve this collaboration. Members of chromatin‐modifying complexes possess “reader” domains that detect and are recruited to specific post‐translational modifications. For instance, bromodomains and YEATS domains show affinity for acetylated lysines while chromodomains, BAH domains, Tudor domains, and MBT domains are recruited to methylated lysines 61, 62, 63, 64, 65. In addition, chromatin complexes can physically interact with other chromatin modules or transcription factors. The NSL complex uses both of these strategies to collaborate with other nuclear proteins to regulate transcription.

The NSL complex consists of a number of “reader” modules (Fig 2A). These modules are particularly enriched in PHF20, which contains a PHD domain as well as two Tudor domains (Fig 2A, F and G). The PHD domain of PHF20 shows specificity for H3K4me2 57, a mark enriched at active promoters and laid down by the MLL/COMPASS family of proteins. Structural and biochemical studies have shown that the E662 residue of PHF20 is critical for the detection of and affinity to H3K4me2 (Fig 2G). Knockdown of *PHF20* in human lung adenocarcinoma cells leads to reduced cell proliferation as well as diminished H4K16ac 57, suggesting that the MOF‐NSL complex is recruited by PHF20 to H3K4me2‐positive promoters where it catalyzes H4K16ac. There is also some evidence that the NSL complex can reciprocally promote H3K4me2. At the *ANKRD2* promoter, depletion of *KANSL1* or *MOF* results in reduced H3K4me2, while depletion of the MLL complex member *RBBP5* only diminishes H3K4me2 and not H4K16ac 66. Thus, there is accumulating evidence that the NSL and MLL complexes not only share components such as WDR5 and HCF1, but they can also corporate at the level of chromatin. However, more work is needed to systematically determine precisely how the crosstalk between the NSL and MLL/COMPASS complexes takes place.

In addition to the PHD domain, PHF20 also possesses two Tudor domains, which are thought to recruit the NSL complex to estrogen receptor alpha (ERα) target genes. Upon activation, ERα is dimethylated by EHMT2 (also known as G9a) at K235 and monomethylated at K303 67. The ERα‐K235me2 modification is recognized by the second Tudor domain of PHF20 (Fig 2F) and is required for the deposition of H4K16ac at ERα/PHF20 target loci 67. Similarly, the second Tudor domain of PHF20 is also able to recognize p53 methylated at K370 and K382 68. The association of PHF20 with methylated p53 is thought to protect p53 from ubiquitin‐mediated degradation and thereby stabilize p53 in response to stress 68. Consistently, depletion of *PHF20* leads to reduced levels of p53 and attenuated activation of p53 target genes. Thus, the NSL complex is able to recognize post‐translational modifications via PHF20 to integrate cell signals with transcriptional output.

The NSL complex can also interact with a number of nuclear proteins. Two independent studies have shown that NSL complex members can physically interact with components of the nuclear pore. The nuclear pore mediates the transport of molecules in and out of the nucleus, and more recent studies have also implicated the nuclear pore complex in regulating chromatin structure 69. Capelson and co‐workers undertook an unbiased comparison of the nuclear pore component NUP98 ChIP profile to other publically available chromatin datasets and found NUP98 to most closely overlap with NSL component MBD‐R2 in *Drosophila* S2 cells 70. Further interrogation of this relationship revealed that MBD‐R2 could immunoprecipitate NUP98. Similarly, another nuclear pore component, NUP153, was found to interact with NSL1 in an unbiased mass spectrometry analysis 34. While the complete significance of these interactions remains to be established, depletion of NSL complex member MBD‐R2 via RNAi leads to reduced recruitment of NUP98 to chromatin 70, suggesting that the NSL complex may be important for the chromatin targeting of nuclear pore components. This is consistent with the observation that the nuclear pore complex is important for chromatin organization 69.

In addition to components of the nuclear pore complex, ChIP profiles display a strong overlap of NSL1 with components of the *D. melanogaster* trithorax and NURF complexes 50. Recent work has identified that NSL complex components interact directly with the NURF complex members NURF301, NURF38, and p55 58. NSL3 was shown to bind TATA‐less housekeeping promoters in a DNA sequence‐dependent manner and the NSL complex was subsequently able to recruit the NURF complex 58. Consistently, depletion of NSL complex members by RNAi leads to disruption of the nucleosomal organization around the transcription start sites of NSL‐bound genes and increased transcriptional noise 58. Thus, NSL‐dependent recruitment of the NURF complex appears to be important for faithful transcription from NSL target housekeeping genes, at least in *Drosophila*. Whether this interaction is conserved in mammalian cells remains to be ascertained.

While the NSL complex can directly bind to DNA at least in *Drosophila*, reports in the mammalian system have perpetrated NSL complex members to interact with DNA‐binding transcription factors, thus providing a potential mechanism for NSL recruitment to chromatin. During osteoblast differentiation, PHF20 upregulates the transcription of *RUNX2*, an important transcription factor in the osteogenic lineage 71. Furthermore, overexpression studies showed that PHF20 and RUNX2 physically interact and synergistically promote transcription at target promoters 71. Similarly, upon activation of c‐Jun in 293T cells, activated but not inactive c‐Jun was able to associate with KANSL2 and KANSL3 but not MSL1 or MSL2 72. The NSL complex was required for mediating the removal of the inhibitory NuRD complex at c‐Jun target loci, increasing H4K16ac and thus inducing the expression of c‐Jun target genes 72. Thus, it is starting to become apparent that the NSL complex binds to promoters of genes and coordinates the activation of transcription together with a number of transcription factors and chromatin remodelers. Since every cell type has unique requirements for the activation of specific genes, it will be important to determine precisely how the NSL complex is recruited in different cell types, whether there is a conserved recruitment mechanism, or whether there are specific binding partners of NSLs in particular cell types.

## The NSL complex beyond chromatin

While the NSL complex is highly enriched at chromatin, recent studies have shed light on the dynamic sub‐cellular localization and function of NSLs, thereby pointing toward a role beyond nuclear gene expression. Members of the NSL complex, KANSL1, KANSL3, and MCRS1 relocate from nuclear chromatin to the minus‐ends of microtubules when cells leave interphase and enter mitosis 73. Using the Xenopus egg extract system, in which transcription is fully inhibited, Meunier and co‐workers showed that KANSL1, KANSL3, and MCRS1 stabilize microtubules and interact with the essential spindle assembly factor TPX2 in a transcription‐independent manner (Fig 3C). The interaction between the NSL complex proteins and TPX2 promotes microtubule assembly and stabilization of chromosomal microtubules. Consistently, knockdown of *KANSL1*,* KANSL3,* or *MCRS1* in HeLa cells results in dramatic mitotic defects including mitotic delay, chromosome scattering, multipolar spindles, and reduced microtubule stability 73. While this study highlights a key role for NSL complex members during mitosis, future studies are required to determine whether this mechanism is conserved in non‐cancerous cells and *in vivo*.

In addition to microtubules, the presence of MOF, KANSL1, and KANSL3 has also been reported in mitochondria 74. MOF and KANSL3 localize to the D‐loop of mitochondrial DNA and are required to maintain transcription of the mitochondrial genome, at least in the context of HeLa cells (Fig 3D). As a consequence, depletion of *MOF* or *KANSL1* in HeLa cells results in impaired mitochondrial respiration 74. The importance of mitochondrial localization of MOF was revealed through rescue studies. Mitochondrial respiration of *MOF*‐depleted cells could be rescued by the reintroduction of a mitochondrially targeted wild type but not catalytic dead MOF, possibly decoupling the nuclear and mitochondrial functions of the MOF‐NSL complex. The role of MOF in the mitochondria is however only beginning to be understood, and several aspects still need to be thoroughly investigated. For instance, since NSL proteins contain both nuclear and mitochondrial targeting signals, it is important to determine the triggers that dictate mitochondrial versus nuclear recruitment of NSL complex members. Furthermore, the knowledge of how MOF depletion from one cellular compartment affects the function of the other organelle would unveil whether and how MOF can mediate cross‐organellar communication to maintain cellular homeostasis. Despite these outstanding questions, the role of MOF in regulating aspects of metabolism is likely to be important. Mouse models lacking *Mof* in cardiomyocytes display swollen and degenerated mitochondria, reduced cardiac function, and eventually die due to cardiomyopathy 74. Furthermore, duplications in exons 1–3 of the human *KANSL1* gene are associated with a greater risk of congenital heart defects 75. It remains to be determined, however, whether the nuclear or mitochondrial function of the MOF‐NSL complex is more important for cardiac development and function.

In addition to regulating metabolism and cell growth, members of the NSL complex have also been implicated in autophagy. mTORC1 is a central regulator of cell growth versus catabolism. mTORC1 promotes cellular growth when nutrients are plentiful while inhibition of mTORC1 is associated with autophagy 76. MCRS1 is required for amino acid‐dependent mTORC1 activation in HEK293T and HCT‐116 cells. GTP‐bound Rheb directly stimulates the autophagy signal integrator mTORC1. In the presence of amino acids, MCRS1 bridges the interaction between Rheb‐GTP and mTORC1 at the site of late endosomes/lysosomes, resulting in mTORC1 stimulation. In the absence of amino acids, the MCRS1‐Rheb interaction is lost and binding between Rheb and the GTPase TSC2 is favored instead. Consistently, loss of MCRS1 mimics amino acid starvation in both mouse and human cells 77. Thus, it appears that MCRS1 and MOF play distinct roles in autophagy, as MOF‐mediated H4K16ac is downregulated following amino acid starvation in MEFs 78. While it is currently difficult to reconcile the differences between MCRS1 and MOF function in autophagy, it is plausible that the role of MCRS1 in autophagy is distinct from its function in the NSL complex as it also associates with the INO80 complex.

While the studies discussed here are starting to reveal important functions of NSL complex members beyond the level of chromatin, there are still a number of outstanding questions that require attention (see Box 1: In need of answers). For instance, do NSL complex members localize to other organelles beyond the nucleus and mitochondria? Do NSL complex members have unique non‐chromatin functions outside of the core NSL complex? What are the precise targets of NSL complex activity in various organelles and the cytoplasm? Given the widespread nature of lysine acetylation 17, and the importance of NSL function in cells at a multi‐organellar level, it is likely that the NSL complex has widespread targets. The identification of the non‐histone targets will help shed light on the extent of NSL function within cells in the context of transcription as well as other novel regulatory mechanisms.

**Box 1: In need of answers**

- Does the NSL complex have MOF‐independent functions?
- How is MOF shared between MSL and NSL complexes? What factors contribute to its recruitment to one complex versus the other complex?
- What are the cellular acetylation targets of the NSL complex?
- Can the NSL complex catalyze longer –acyl chains such as propionyl or crotonyl?
- How is the NSL complex recruited to chromatin? Does it have unique binding partners?
- What are the H4K16ac‐independent roles of the NSL complex at chromatin?
- How do expression changes in MOF and NSL complex members drive cancer?
- Does the NSL complex regulate metabolic networks and mitosis *in vivo*?
- Does the NSL complex localize to any other cellular organelles? If so, what is the function of NSL proteins there?
- What are the molecular mechanisms underlying intellectual disability and developmental delay observed in human *KANSL1* haploinsufficiency?

## NSL complex in human health and disease

Given the critical role of the NSL complex in maintaining cellular homeostasis, mutations or deregulation of NSL proteins leads to severe human disease. Heterozygous mutations in *KANSL1* have been found in around 1:16,000 live births 79 and underlie the KdV syndrome 80, 81, a severe developmental disorder typified by developmental delay, intellectual disability, facial dysmorphisms, low birthweight, and a range of comorbidities 79. Furthermore, a screen for genetic variants in severe intellectual disability identified mosaic point mutations in *KANSL2*, which are predicted to induce splicing defects in *KANSL2* transcripts 82. The precise frequency of disease‐causing *KANSL2* variants remains to be determined. In comparison, given the high prevalence of KdV syndrome, KdV patients have been well characterized. KdV patients typically present with a low IQ, but display friendly behavior 79. Recent MRI studies revealed that KdV patients generally display morphological defects including hippocampal malformations, corpus callosum dysgenesis, and dilated ventricles 83. Given the severity and prevalence of the KdV syndrome, researchers have attempted to model aspects of KdV in mouse models to determine the underlying molecular mechanisms. *Kansl1* ^+/−^ mice model most aspects of KdV patients, including low body weight and reduced performance in memory and learning tests such as novel object recognition assays and fear conditioning paradigms 84. However, despite these animal studies and analysis of human patients, the underlying molecular networks leading to defective development and brain function in KdV patients remain unknown. Given that *KANSL1* haploinsufficiency likely results in reduced levels of KANSL1 79 and KANSL1 is required for the full catalytic activity of MOF within the NSL complex 43, the underlying molecular defect is likely to involve reduced acetylation levels at NSL targets and disrupted gene transcription. Transcriptomic studies on human patient material and in mouse models are required to determine precisely which gene networks are dysregulated upon *KANSL1* haploinsufficiency.

In addition to developmental disorders, deregulation of NSL complex members has been reported in malignancies (Fig 3E). Reduced H4K16ac is strongly associated with a wide range of malignancies 85. Consistently, independent studies have reported MOF to be downregulated in a panel of cancer types including breast cancer 86, ovarian epithelial cancer 87, colorectal carcinoma 88, gastric cancer 88, 89, hepatocellular carcinoma 90, and medulloblastoma 86. How the downregulation of MOF and H4K16ac drives tumorigenesis remains unknown, as paradoxically, depletion of MOF leads to cell cycle arrest in primary cell culture systems 55.

In contrast to MOF and H4K16ac, other members of the NSL complex appear to play a pro‐oncogenic role in cancer. MCRS1 upregulation has been reported in colorectal carcinoma 91, 92, glioma 93, 94, and NSCLC 93. In each of these cancers, increased levels of MCRS1 correlate with cancer aggressiveness and poor survival. Consistently, knockdown of *MCRS1* in colorectal cancer cells leads to reduced cell proliferation along with lower levels of cyclin D1 and CKD4 as well as increased p21 expression 91. Similarly, *MCRS1* depletion in glioma cells results in reduced tumorigenicity both *in vitro* and in nude mouse models 94. Reminiscent of MCRS1, KANSL2 is reportedly upregulated around sevenfold in glioblastoma samples compared to adjacent normal tissue 95. KANSL2 was shown to drive the stem cell‐like features of glioblastoma cells, and knockdown of *KANSL2* reduced expression of the pluripotency factors *NANOG and POU5FI,* while it increased the expression of the neural differentiation markers *TUBB3* and *GFAP* 95. Consistently, reduced *KANSL2* levels result in smaller tumors after transplantation of KANSL2‐depleted glioblastoma cells into immunodeficient mice.

There is one notable exception to the downregulation of MOF in cancers. MOF is highly expressed in non‐small‐cell lung cancer (NSCLC), and increased expression levels are associated with poor survival 96. In contrast, reduced *PHF20* levels are reportedly associated with poor survival in NSCLC 97. The underlying reason for this unique correlation between MOF, PHF20 levels, and NSCLC progression is unknown, but it will be important to determine, as NSCLCs account for around 85% of lung cancers and are typically resistant to chemotherapy.

It is interesting to note that reduced MOF and H4K16ac levels but increased levels of MCRS1 and KANSL2 are associated with cancer progression and aggressiveness. While these observations are difficult to reconcile on a molecular level, it is plausible that reduced MOF levels in cancer are associated with the activity of the MSL complex. Consistently, depletion of MSL complex but not NSL complex members is associated with a global reduction of H4K16ac in both *Drosophila* and mammals 35, 47, 48, 56, which is similar to the observations in cancer cells 85. In the future, it will be important to determine the function of MOF in the context of the MSL versus NSL complex in driving malignancies. Moreover, further molecular characterization should reveal important insights into the functions of the two complexes in driving cancer traits such as self‐renewal, cell proliferation and apoptosis.

Among NSL complex members driving cancer traits, mutations in *KANSL1* have been reported to promote malignancies. Zhou and co‐workers recently identified a *KANSL1‐ARL17A* fusion transcript in cancer samples isolated specifically from patients with European ancestry 98. *KANSL1* and *ARL17A* are neighboring genes on human chromosome 17. The authors found that the *KANSL1‐ARL17A* fusion is inherited within European populations and could predispose carriers to cancer 98. In addition, *KAT6B‐KANSL1* translocations have been identified in a woman with retroperitoneal leiomyoma 99, but how the *KAT6B‐KANSL1* translocation drives this cancer remains to be identified. It is likely that with more widespread use of next‐generation sequencing technologies, novel mutations in NSL complex members in cancer will be identified. In addition to these mutations, NSL and MLL complex members have been shown to interact with leukemogenic translocation proteins NUP98‐HOXA9, NUP98‐HOXD13, NUP98‐NSD1, NUP‐PHF23, and NUP98‐TOP1 100. These findings are consistent with work showing that NSL complex members associate with wild‐type nuclear pore complex components 34, 70. Xu and co‐workers found that depletion of *Mll1* from NUP98‐HOXA9 driven leukemic cells was sufficient to reduce the number of leukemic cell growth and increase survival of mice transplanted with NUP98‐HOXA9 transduced hematopoietic progenitor cells 100. The significance of the NSL complex interaction with NUP98 fusion proteins was not determined in this study, and whether the NSL complex is required for the NUP98 fusion protein‐driven leukemic transcription program remains to be established.

Together, these studies suggest an important role for the MOF‐NSL complex in regulation of neurological functions and carcinogenesis. Future studies are required to better establish the molecular mechanism by which the individual NSL complex members orchestrate neural development and regulate tumor growth, metastasis, and invasion. Given the variable requirements for NSL complex members for growth of different cancer types, it will be important to study NSL proteins in specific cancer subtypes to better understand their role in cancer.

## Conclusions

Since the discovery of the NSL complex just over a decade ago, a number of studies spanning mouse and *Drosophila* model systems as well as human samples have revealed the functional importance of the NSL complex (Fig 3). At the level of chromatin, the NSL complex regulates transcriptional networks critical for the maintenance of cellular homeostasis. Mutations or deregulation of NSL complex members results in malignancy or developmental disorders. In addition, the individual complex members are beginning to be biochemically characterized and their catalytic activities and binding partners are being established. We are now also starting to appreciate that the NSL complex functions beyond mere transcriptional regulation. Certain NSL complex members localize to microtubules as well as to the mitochondria in a dynamic manner. Whether NSL complex members can also localize to other organelles remains to be determined and will be an exciting avenue of research to pursue. Furthermore, whether NSL complex members show dynamic cell type‐dependent recruitment to chromatin or to different organelles remains to be established. Work utilizing *Mof*‐knockout mouse models 51, 55, 78, 101, 102, 103 indeed suggests that the NSL complex is likely to possess cell type‐specific and cellular context‐specific functions, which remain to be formally tested in NSL model systems. Furthermore, global screens for MOF‐NSL acetylation targets as well as NSL binding partners in different cell types are required to better understand the extent of NSL‐mediated control in cells (see Box 1: In need of answers).

Overall, it is becoming clear that the NSL complex plays a central role in controlling transcriptional networks and cell signaling pathways and is deregulated in a range of human disease (Fig 3). Thus, it is pertinent to better understand the mechanistic workings of the NSL complex. The field will look toward developing a better understanding of the myriad of NSL complex functions as well as the biochemical role of its individual members in the coming years.

## Conflict of interest

The authors declare that they have no conflict of interest.
